# High mobility group motif proteins’ role in fibrosis, inflammation, and vascular injury in systemic sclerosis

**DOI:** 10.1007/s00109-026-02679-5

**Published:** 2026-06-10

**Authors:** Fabian A. Mendoza, Sonsoles Piera-Velazquez, Sergio A Jimenez

**Affiliations:** 1 Jefferson Institute of Molecular Medicine and Scleroderma Center, Thomas Jefferson University, Philadelphia, PA 19107 USA; 2 Division of Rheumatology, Department of Medicine, Thomas Jefferson University, Philadelphia, PA 19107 USA; 3 Department of Dermatology and Cutaneous Biology, Thomas Jefferson University, Philadelphia, PA 19107 USA

**Keywords:** Scleroderma, Systemic sclerosis, HMGB, SOX9, Endothelial dysfunction, Micro-vesicles, Fibrosis, Damage-associated molecular patterns, TGF-beta

## Abstract

Systemic Sclerosis (SSc) is an idiopathic systemic autoimmune disease characterized by progressive cutaneous and systemic fibrosis, severe vasculopathy, and multiple humoral and cellular immunological alterations. The pathogenesis of SSc is highly complex and remains incompletely elucidated. The fibrotic process is a crucial component of SSc and is responsible for organ failure and high mortality. Although an increasing understanding of the fibrotic process has enabled the clinical development of antifibrotic therapeutic agents, these agents have limited clinical efficacy. Recently, the potential role of a group of transcription factors containing a High Mobility Group (HMG) motif, in the development and pathological manifestations of SSc has been postulated. HMG proteins (notably HMGB1 and SOX9) act as profibrotic and proinflammatory transcription factors; however, HMG proteins can also function as damage-associated molecular patterns, amplifying Toll-like receptors and RAGE signaling, promoting endothelial activation, leukocyte recruitment, and stimulating the production of profibrotic cytokines. This convergent role of HMG proteins across various aspects of SSc pathogenesis, including immune dysregulation, vasculopathy, and fibrosis, makes them among the most attractive novel regulators and a desirable therapeutic target for SSc. Here, we review the recent evidence on the role of HMG proteins in SSc pathogenesis and explore the potential role of inhibiting their function.

## Introduction

Systemic Sclerosis (SSc) is a systemic autoimmune disease of unknown etiology characterized by cutaneous and internal organ fibrosis, associated with a severe vasculopathy that presents as Raynaud’s phenomenon, telangiectasias, and vascular proliferative and occlusive lesions. The SSc internal organ involvement is responsible for the high mortality of the disease. It manifests most commonly as esophageal and gastrointestinal dysfunction, pulmonary fibrosis, pulmonary arterial hypertension, cardiac fibrosis, and renal involvement that frequently presents as renal crisis [1–3]. Early diagnosis of SSc before evidence of internal organ damage is of great importance, as it may lead to improved clinical outcomes, less severe internal organ involvement, and reduced mortality [4]. Currently, there is no curative therapy for SSc, and the development of an effective therapeutic approach requires further elucidation of its etiology and pathogenesis.

Although the etiology of SSc remains unknown, it has become increasingly clear that a significant genetic component contributes to the initiation and clinical progression of the disease [5–8]. A close interplay between this genetic component and environmental factors triggers multiple immunological alterations, endothelial dysfunction, and a pro-fibrotic state. Recent evidence shows that these processes are interconnected. This exceptionally complex pathogenesis [9–13] results in highly variable symptoms and signs, a heterogeneous clinical course, and multiple outcomes, including a high mortality rate. However, there is a growing consensus that vasculopathy and endothelial cell abnormalities are crucial to SSc, and it is well recognized that severe vascular remodeling and occlusion, particularly involving the microvasculature, play a pivotal role in the development and progression of tissue inflammatory changes and fibrosis [14–16].

**Fig. 1:**
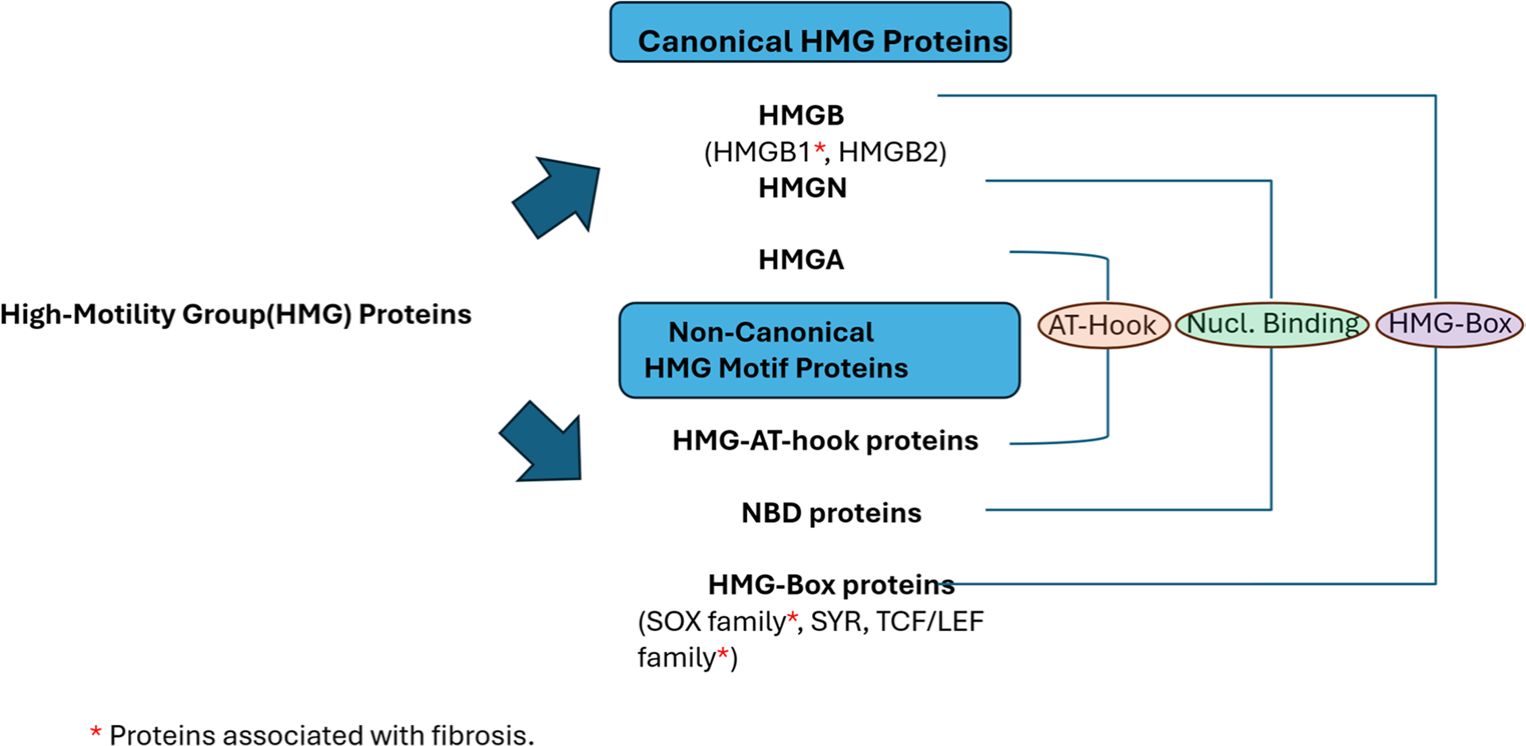
HMG group proteins. Transcription factors that share the high-mobility group (HMG) domain can be classified as canonical or non-canonical proteins. Both groups share binding domains such as the AT-hook, nuclear binding, and HMG-box. This figure illustrates this classification. Proteins marked with a red asterisk are found to have strong evidence of a pro-fibrotic role

The multifactorial pathogenesis of SSc, combined with the limited understanding of its etiology, presents substantial challenges for both diagnosis and treatment of the disease. However, there has been recent interest in the role of the High Mobility Group (HMG) motif proteins, a group of transcription factors characterized by their high electrophoretic mobility, in SSc pathogenesis. HMG proteins are the largest group of nuclear proteins after histones. Based on their functional domains and structures, these proteins are divided into three (canonical) families: HMGA (formerly known as HMGI/Y), HMGN (or HMG14/17), and HMGB1-4 domain proteins (previously referred to as HMG1/2). These proteins contain an AT-hook, nucleolar-binding, or HMG-box functional motifs, respectively [17–21]. These functional motifs are also shared in a wide range of eukaryotic (non-canonical) proteins. In humans, the family of proteins sharing the HMG-box motif includes the SOX and the TCF/LEF family (also known as the T cell factor/lymphoid enhancer factor family) [22]. These proteins play multiple and diverse physiological roles in DNA transcription. Recently, several members of the extended HMG-box family have been shown to stimulate the synthesis of various macromolecules involved in pathological fibrosis [23–32]. Additionally, some of its members can also be secreted through microparticles, leading to endothelial damage and tissue inflammation. Therefore, these proteins may play multiple roles in novel molecular mechanisms underlying the pathogenesis of SSc and other fibrotic diseases, including pulmonary, hepatic, and renal fibrosis. A schematic representation of the family of HMG proteins and their members associated with fibrosis is shown in Fig. 1. In this review, we will focus on proteins that share the HMG-box domain (HMGB and HMG-box proteins), given the extensive recent body of evidence supporting their pathogenic role.

## HMGB transcription factors

**Fig. 2:**
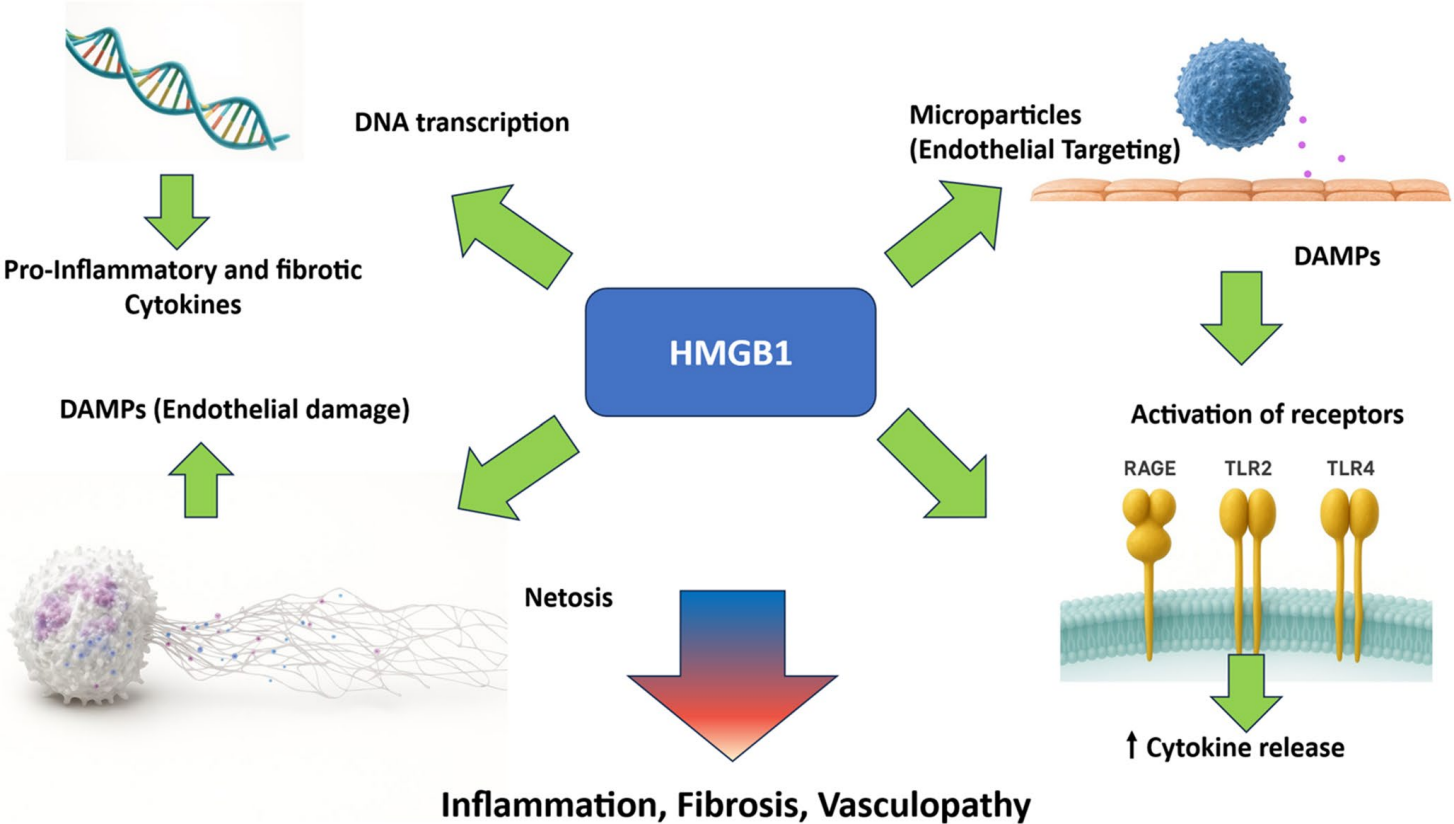
HMGB1 Pleotropic roles. HMGB1 plays different roles depending on its context, such as its intracellular (or extracellular) location, redox state, and pH. In the cell nucleus, it participates in the transcription of various pro-inflammatory and pro-fibrotic factors; in the cytoplasm, it can regulate mitochondrial function and apoptotic pathways (not shown); and it can either be secreted into microparticles that target endothelial cells or expelled via Neutrophil Extracellular Traps (NETs). Extracellular HMGB1 can act as a DAMP, causing endothelial damage, and can also stimulate Toll-like Receptors (TLRs) and RAGE, thereby triggering the release of pro-fibrotic cytokines. Taken together, HMGB1 can activate pro-inflammatory and pro-fibrotic pathways and promote endothelial dysfunction; all of which play a crucial role in SSc pathophysiology

The members of the HMGB subfamily are the most extensively studied proteins within the HMG protein family. These proteins are characterized by the presence of one or more motifs, each of approximately 80 amino acids long, termed the HMG box [19, 20], which demonstrate non-sequence-specific DNA binding [33, 34]. However, they are able to recognize and bind to structurally disordered DNA regions [35]. HMGB1 and HMGB2 are actively involved in transcription, replication, and DNA repair [33–35]. In addition, other non-canonical HMG proteins displaying the HMG-box motif share DNA-binding properties with the above-mentioned transcription factors [36, 37] (Fig. 2).

**Fig. 3:**
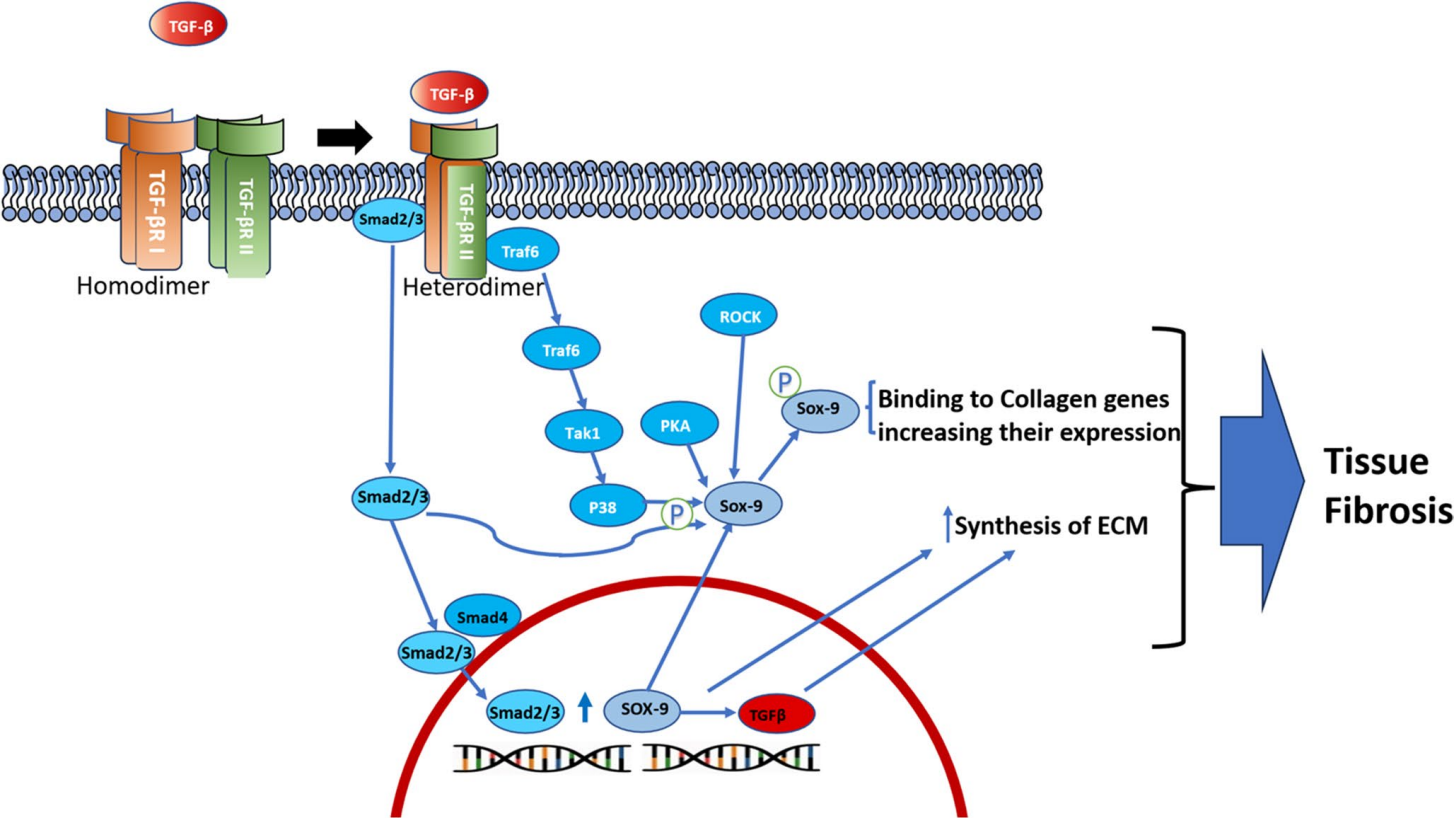
Regulation of SOX9 expression by TGF-ꞵ. Following release from its complex, TGF-β binds its receptor, leading to heterodimerization and activation of the TGF-β receptor. This activation triggers the canonical pathway by phosphorylation of Smad2 and Smad3. Those intracellular mediators form a complex with Smad4, allowing its translocation through the nuclear membrane. Smad2/3 increases the transcription of SOX9. SOX-9 mediates the increase in the transcription of genes encoding ECM and also increases the transcription of TGF-ꞵ2, thereby promoting tissue fibrosis. On the other hand, Smad3 and non-Smad TGF-β intracellular mediators, such as Traf6, Tak1, and p38, phosphorylate SOX9. Subsequently, SOX9 regulates collagen gene expression by direct binding to specific intron sequences. These combined effects of TGF-β and SOX9 result in increased tissue fibrosis

HMGB1 and HMGB2 are evolutionarily conserved proteins with very similar amino acid sequences and structures. Both consist of a short N-terminal region, two DNA-binding domains, and a C-terminal region of 20–30 amino acids. It is in this terminal region that they minimally differ in the sequence of glutamic and aspartic acids, but the minimal differences in sequence and the importance of the negatively charged C-terminal sequences in the interaction with proteins and nucleic acids suggest that differences in the pattern of their post-translational modifications and 3D structural organization may explain their functional differences [35–37] (Fig 3).

**Fig. 4:**
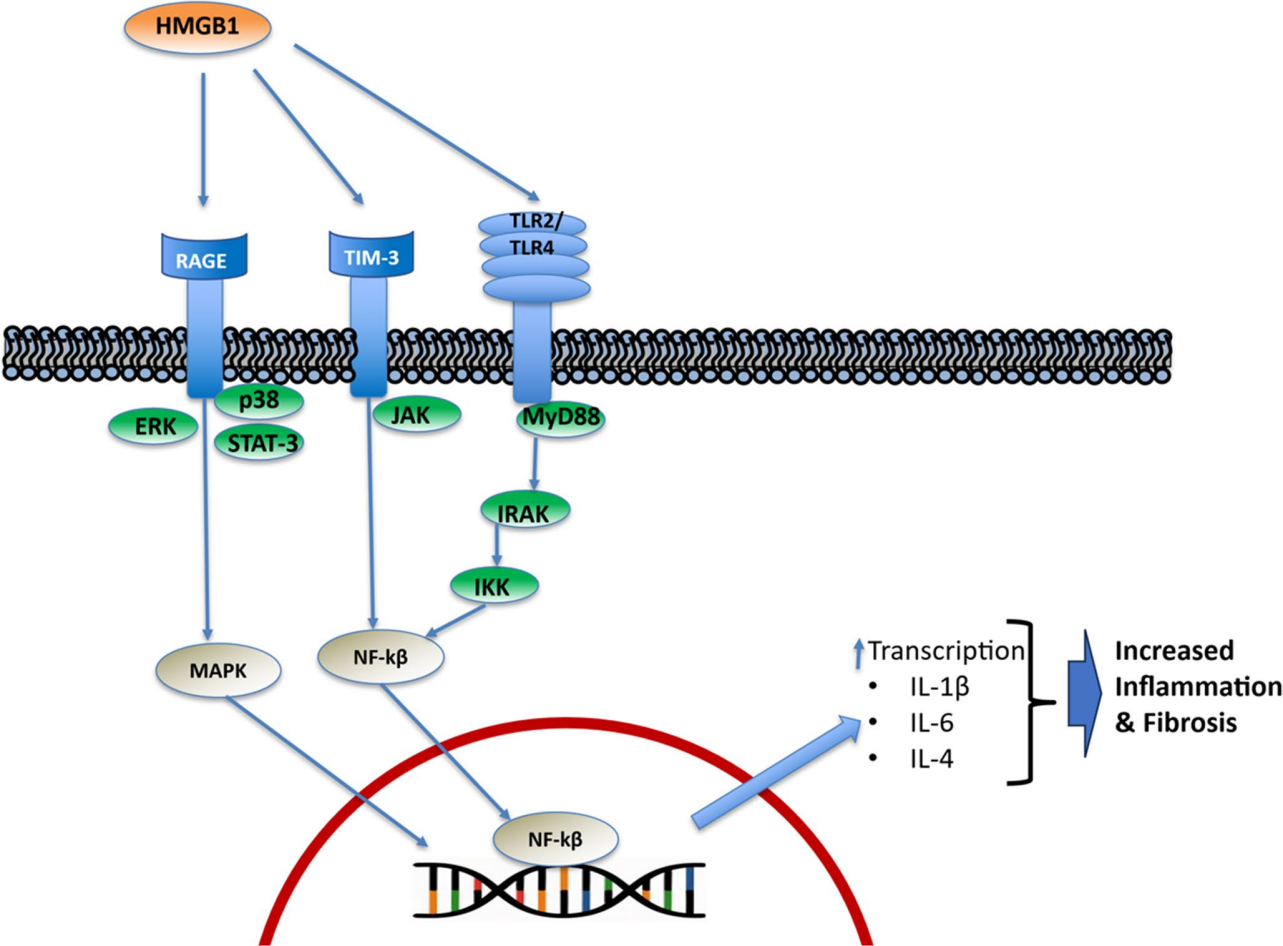
Extracellular effects of HMGB1 in its cellular receptors. Following its release, HMGB1 can migrate inside microparticles toward its effector cells and can link to multiple receptors, including but not limited to TLR2/TLR4, RAGE, and TIM3, triggering multiple intracellular pathways, including MAPK, ERK, and NFK-β, promoting inflammation and a pro-fibrotic state through the expression of cytokines such as IL-6, IL-4, and IL-1

The variety of functions performed by these proteins is associated with their localization in the cellular/intercellular space [19, 38–40]. Depending on its post-translational modifications (PTMs) and redox status, HMGB1 can leave the nucleus, translocate into the cytoplasm, and exit to the intercellular space, where it can function as a signaling molecule in response to damage to cell integrity and necrosis. HMGB1 is subject to post-translational modifications such as phosphorylation, methylation, acetylation, glycosylation, and poly-ADP-ribosylation [40]. Whereas phosphorylation and acetylation are needed for its interaction with DNA, oxidation of cysteine residues in the HMG-box domain, acetylation, and ADP-ribosylation determine its intracellular translocation [40]. Additional Redox changes and the formation of disulfide bridges drive the protein secretion into the extracellular space. In the extracellular space, HMGB1 can (1) activate MAPK-associated receptors [41], triggering MAPK and NF-kB activation [42], propagating tissue damage, and (2) activate the receptor for advanced glycation end products (RAGE), a transmembrane multi-ligand receptor located in the vicinity of the HLA complex [43]. Expression of RAGE is dramatically induced in type I alveolar epithelial cells (AECI) and infiltrated inflammatory cells [44]. Mouse models suggest that HMGB1 Signaling through RAGE contributes to the secretion of pro-inflammatory cytokines, but these pathways may require co-stimulation through TLR2 and TLR4 [45]. In addition to direct receptor interaction, HMGB1 may form heterocomplexes with other molecules, such as interleukin-1 (IL-1) and CXC ligand 12 (CXCL12). When forming a complex with CXCL12, HMGB1 promotes CXC receptor type 4 (CXCR4)-dependent recruitment of inflammatory cells to affected tissues [46]. A summary of HMGB1 pleotropic effects is displayed in Figs. 2 and 4.

In contrast, HMGB2’s intracellular and extracellular roles remain largely unexplored. However, a recent study focused on HMGB2 modifications and secondary structures has analyzed and highlighted their functional divergence from HMGB [35]. Furthermore, recent evidence indicates that HMGB2 has an important role in mediating the senescence-associated secretory phenotype (SASP), a hallmark of senescence with relevant physiological effects, linking senescence to inflammation. Effectively, chromatin-bound HMGB2 fine-tunes SASP expression by avoiding heterochromatin spreading and upregulates SASP-related genes such as IL-8 and IL-6 [47]. This recent discovery opens the possibility of inhibiting epigenomic regulation of the SASP and suppressing the inflammatory component of senescence. Emerging evidence suggests that cellular senescence may be a key factor in SSc progression. For instance, oxidative stress and viral infections such as parvovirus B19, have been shown to induce cellular senescence in human dermal fibroblasts, which may contribute to SSc-associated fibrosis [48, 49].

## SOX9 transcription factor

SOX9 is a member of the SRY-related high mobility group (HMG) family of multifunctional transcription factors that plays a crucial role in organogenesis and development [50–60]. SOX9 contains an N-terminal self-dimerization region followed by the DNA-binding HMG domain and central and C-terminal transactivation domains. SOX9 is a pioneer transcription factor with the capacity to bind regions of closed or condensed chromatin, resulting in enhanced accessibility to genes required for cellular differentiation [53].

SOX9 exerts marked effects on chondrocyte differentiation and chondrogenesis, including induction of mesenchymal condensation into chondrocytes [50–52]. SOX9 also regulates male sexual development [54] and is essential for the development of multiple organs, including bone, testis, heart, lung, pancreas, intestine, and nervous system [54, 55]. SOX9 exerts these effects by directly regulating the transcription of genes encoding extracellular matrix components, including Collagens Type II, Type IX, and Type XI. Mutations in the human SOX9 gene have been described in several diseases, including Campomelic Dysplasia, a haploinsufficiency disorder with multiple skeletal malformations, and tracheomalacia, which is frequently accompanied by 46, XY sex reversal [57, 58].

## Role of extracellular HMGB1 in SSC inflammation

Inflammation and immune dysregulation are two of the main pathophysiological processes involved in the early development of SSc. Specifically, innate and acquired immune dysregulation can play a role in early SSc events. In this context, NETosis (Neutrophil extracellular traps -NETs- activation) may promote tolerance breakdown by self-antigen exposure, and by direct endothelial damage, causing widespread endothelial dysfunction and vasculopathy [59].

A recent study has demonstrated that platelet-derived microparticles in the serum of SSc patients have a markedly higher HMGB1 concentration than those from healthy individuals or patients with systemic lupus erythematosus (SLE). These HMGB1-rich microparticles interact directly with co-cultured neutrophils from healthy donors, triggering autophagy, activating fibrinolysis, and inducing NET release [61]. These effects in in-vivo models are significantly reduced by BoxA, a specific HMGB1 antagonist, suggesting that HMGB1 is a central effector molecule in SSc pathogenesis [60].

## Role of HMGB1 and SOX9 in tissue fibrosis

HMGB1-rich microparticles, isolated from SSc patients, promoted neutrophil autophagy when injected into immunocompromised mice, as described above. However, they also induced neutrophil migration within the lung tissues, leading to abnormal collagen accumulation in the interstitial space. In animal models of SSc, such as TSK mice or those induced by bleomycin, increased serum HMGB1 levels were observed compared with controls [62, 63]. A similar increase in HMGB1 serum levels is also observed in serum from SSc patients compared with healthy controls [62]. HMGB1 concentration is not only higher in patients with pulmonary fibrosis, digital ulcers, abnormal nailfold capillaroscopy, and arthritis, but also correlates directly with the modified Rodnan skin score (mRSS) and inversely with the expected percentage of the forced vital capacity (FVC) [61, 64]. Other studies have also shown a relationship between serum HMGB microparticle concentration and the presence of ILD in SSc [64].

Another recent study correlated increased HMGB1 levels with elevated serum calpain, a TGF-β co-mediator of epithelial-mesenchymal transdifferentiation (EMT), a key phenomenon in the development of pulmonary fibrosis that has also been demonstrated in the fibrotic skin of SSc patients [65].

At the transcriptional level, recent studies have demonstrated that the HMG-box motif protein SOX9 regulates the expression of various collagens and other proteins involved in the fibrotic process, suggesting its potential role in the pathogenesis of diseases associated with tissue fibrosis [27–29]. Indeed, it has been postulated that SOX9 plays a crucial role in lung, kidney, and liver fibrosis [30, 32, 66–69]. SOX9 also accelerates vascular aging [70], and recently, it has been suggested that alterations in genes encoded by the SOX9 locus may also contribute to SSc pathogenesis [71]. Our recent studies analyzed dermal fibrosis samples from SSc patients using RNA sequencing. They demonstrated increased SOX9 phosphorylation in SSc dermal fibroblasts compared with controls, supporting the involvement of SOX9 in the altered gene expression observed in the disease (Piera-Velazquez et al., In-Press). Given the critical role of endothelial cells in SSc pathogenesis, a recent study demonstrated that the ability of endothelial cells to induce tissue fibrosis may be related to their potent effects on activating SOX9 [72].

Other transcription factors that carry the HMG motif also play a role in fibrosis, but they have not been extensively studied. For example, the TCF/LEF transcription factor binds with beta-catenin after beta-catenin enters the nucleus and mediates Wnt-beta-catenin pro-fibrotic gene transcription [73]. Canonical Wnt signaling has been recently characterized as a key mediator of sustained fibroblast activation in SSc [74].

## Regulation of expression of HMG proteins by TGF-β

TGF-β is considered the master regulator of fibrosis [75–77], and it has been suggested to play a crucial role in SSc pathogenesis (Reviewed in Ref. [78]).

Several members of the extended HMG family have been demonstrated to interact with TGF-β during the development of fibrosis.

A transcription factor, member of the HMG family with the AT hook 2 (HMGA2), is crucial for endothelial-mesenchymal transdifferentiation (EMT). For example, in tissues from patients with idiopathic pulmonary fibrosis (IPF) and in lung cells (A549 and human bronchial epithelium), HMGA2 expression was increased, whereas miR-221 expression was inhibited [79]. Transfection models demonstrated that miR-221 inhibits bleomycin-induced pulmonary fibrosis by suppressing HMGA2 expression, which in turn regulates TGF-β-mediated epithelial-to-mesenchymal transition (EMT) through Smad3 [25, 79]. Another member of the same sub-family, HMGA1, acts as a transcription factor in several cardiovascular diseases. In a mouse model of cardiac fibrosis, it was shown that HMGA1 was upregulated in fibrotic hearts [80].

Overexpression of HMGA1 transcription factor aggravated TGFβ1-induced fibroblast activation, proliferation, and collagen accumulation. A viral transfection model overexpressing HMGA1 accelerated cardiac fibrosis and cardiac dysfunction; furthermore, HMGA1 knockdown inhibited the profibrotic effects of TGFβ1. These pro-fibrotic effects appear to be mediated through an increased transcription of fibroblastic FOXO1 [80].

TGF-β also seems to regulate other HMG transcription factors. Furthermore, we recently performed gene expression analysis of SSc skin and demonstrated the concomitant elevation of phosphorylated SOX9 and TGF-β in affected SSc dermal tissue, suggesting that SOX9 and TGF-β act together in a complex regulatory interplay integral to SSc pathogenesis (Jimenez and Piera-Velazquez: submitted to publication). Moreover, there is experimental evidence from studies of malignant diseases such as gliomas [81] and gastric cancer [82], as well as from chondrocytes [83], indicating that TGF-β stabilizes SOX9 and thereby increases SOX9 activity.

TGF-β-stabilizing SOX9 is also pertinent to the development of pulmonary fibrosis [84]. Furthermore, SOX9-mediated upregulation of TGF-β in pulmonary fibroblasts may contribute to additional SOX9-mediated upregulation of other molecules participating in the fibrotic process, including COL3A1 and COMP. A similar interplay between SOX9 and TGF-β was observed in fibroblasts isolated from experimentally induced atrial fibrosis [85]. Moreover, in this model, SOX9 inhibition using siRNA decreased the expression of myofibroblast markers, including α-smooth muscle actin (α-SMA). These observations are highly relevant, as myofibroblasts are the keystone effectors in the pathogenesis and maintenance of SSc tissue fibrosis.

The mechanisms regulating SOX9 gene activation are extremely complex and have not been fully elucidated. However, current evidence indicates a close interplay with TGF-β. Indeed, it has been shown that TGF-β receptor activation causes phosphorylation of Smad2 and Smad3. This canonical pathway can promote the transcription of collagen and ECM molecules after Smad2/3 are translocated into the nucleus by Smad4. However, Smad2/3 can also directly increase SOX9 transcription. Conversely, SOX9 mediates increased transcription of genes encoding ECM molecules and can also increase transcription of TGF-1 [86]. These combined effects result in increased extracellular matrix (ECM) accumulation and tissue fibrosis. In addition, activation of other receptors, such as insulin-like growth factor-II (IGF-II), which is overexpressed in lung tissues and fibroblasts in SSc patients, also induces SOX9 expression. This complex interplay is illustrated in Fig. 3. In addition to TGF-β, other molecules also regulate SOX9 gene activation, and the mechanism appears to be highly complex. For example, calmodulin (CaM) has been shown to modulate SOX9’s transcriptional activity, mainly by facilitation of CaM-dependent Sox9’s nuclear import [87, 88]. This interaction is mediated by high and low-affinity nuclear localization sequence domains termed bromodomains [88]. Bromodomains (BRD) are acetyl lysine ‘reader’ modules found in various proteins that mediate protein-protein or protein-peptide interactions. They seem to play a role in stabilizing Sox9 protein. This is supported by the observation that BRD inhibitors, such as JQ1, strongly downregulate SOX9 transcription by interfering with the BRD4-SOX9 protein-protein interaction and thereby affecting protein stability. Still, intriguingly, BRD inhibitors can also affect SOX9 transcription [89] .

Studies of various cancer cells have also identified additional regulators. For example, in esophageal cancer cells, YAP1, a transcription coactivator, activates SOX9 transcription through the YAP1/TEAD complex, and in metastatic cells, SLUG increases SOX9 stability by inhibiting its ubiquitination and subsequent proteasomal degradation [90]. In addition, recent studies from our laboratories demonstrated that the SOX9 gene promoter is activated by Sp1 and CREB [91].

**Fig. 5:**
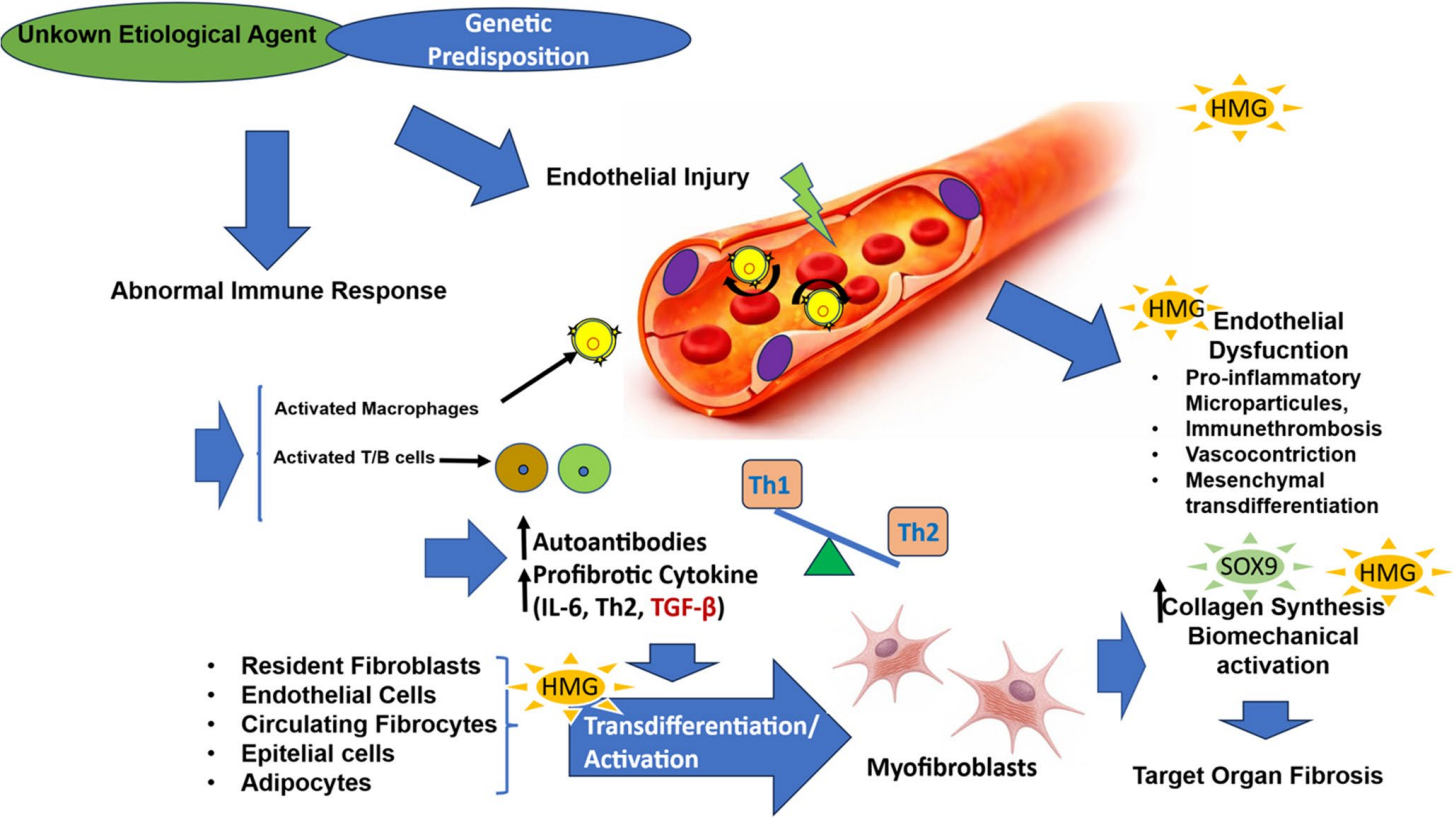
Proposed effects of HMG motif proteins in SSc pathophysiology. Following an unknown initial insult in a genetically predisposed host, (viruses or chemical agents have been proposed), an early inflammatory event and microvascular injury interplay to mount an abnormal immune response with macrophage, T and B cell activation, along with immune mediated endothelial dysfunction. Endothelial cells interact with their surroundings through microparticles, expanding the pro-fibrotic inflammatory phenomena. In addition, a profibrotic cytokine environment, mainly driven by TGF-β will cause mesenchymal trans-differentiation and activation of endothelial, epithelial, adipocytes, circulating fibrocytes and resident fibroblasts into myofibroblasts. Myofibroblasts will cause fibrosis of target organs through an exaggerated collagen production and interaction with its extracellular matrix. HMGB1 plays a role in SSc endothelial damage, causing widespread endothelial dysfunction and vasculopathy through NETosis. SOX-9 induce endothelial cells pro-fibrotic signatures. HMGB1 also promotes the recruitment of inflammatory cells, and induces epithelial-mesenchymal trans differentiation, whereas SOX9 mediates increased transcription of genes encoding ECM molecules and also increase transcription of TGFβ-1

Figure 5 provides an integrated overview of the role of HMG-motif proteins in SSc pathophysiology across different biological events.

## Role of HMGB1 in SSC vasculopathy

As mentioned earlier, vasculopathy is one of the main aspects of SSc pathophysiology. Circulating microparticles secreted by endothelial cells, platelets, and leukocytes, carrying DNA, RNA, pro-inflammatory cytokines, adhesion molecules, and pro-coagulant factors, play a role in SSc endothelial dysfunction and are also potential biomarkers of vasculopathy in SSc [92].

In vivo, SSc microparticles prompt neutrophil migration to the lungs, increase tissue collagen deposition (a hallmark of fibrosis), and elevate levels of soluble E-selectin, a marker of endothelial activation. The microparticles also enhance neutrophil survival in circulation by promoting autophagy, which prevents apoptotic clearance [92]. In contrast, control microparticles from healthy donors or SLE patients do not induce these effects to the same extent. A high concentration of HMGB1 on those particles from SSc patients has been postulated to mediate its inflammatory and pro-fibrotic effects [92].

Furthermore, the blood of SSc patients contains autophagic neutrophils and elevated levels of NET by-products, including citrullinated histones and MPO-DNA complexes. These products correlate with early disease activity and vascular abnormalities, as assessed by nailfold videocapillaroscopy [93]. The same study also found that platelet-derived microparticles (PDµPs) from collagen-activated platelets mimic the effects of SSc microparticles, reinforcing the hypothesis that exposure of subendothelial collagen during vascular injury perpetuates platelet activation and microparticle release [93]. In summary, those authors propose a model in which activated platelets release HMGB1 + microparticles that, in turn, activate neutrophils, rendering them pro-inflammatory and cytotoxic. This process causes neutrophil-mediated endothelial injury, impaired vascular repair, and fibrosis—the hallmarks of SSc pathogenesis. These findings open new avenues for therapeutic intervention targeting the HMGB1-neutrophil axis and suggest that HMGB1+ PDµPs and NET components could serve as biomarkers for disease activity and progression.

No studies have specifically examined the role of SOX9 in SSc vasculopathy. We propose that vessel-specific SOX9 activation may be a crucial molecular mechanism responsible for the severe and often progressive vascular and fibrotic changes in SSc, given that SOX9 activation is a crucial early event in EndoMT trans-differentiation [94, 95].

## Potential inhibition of HMG pro-fibrotic effects

From a review of the studies described above, it has become apparent that many members of the HMG protein family exhibit potent pro-fibrotic effects; therefore, therapeutic agents that prevent or inhibit these effects should be examined as potentially effective therapeutic interventions for wound healing, SSc, and other fibrotic conditions.

1. **Inhibition of HMGB1.**
  As described above, HMGB1 is a nuclear transcription factor that plays many critical intracellular functions but also has extracellular roles. Its role as a transcription factor involves many intracellular processes crucial to life, as evidenced by the finding that its complete knockout results in neonatal lethal hypoglycemia in mice [96]. Even though tissue-specific conditional knockouts have been successfully developed [97], most research on HMGB1 inhibition has focused on extracellular HMGB1 inhibition over the last few decades.
  A. Binding extracellular HMGB1. HMGB1 can also behave as a pathogen-associated molecular pattern (PAMP) when secreted into the extracellular space, activating pattern recognition receptors, including toll-like receptors (TLRs) and RAGE, as explained above. Binding or blocking extracellular HMGB1 can result in a potent anti-inflammatory effect, reducing levels of CRP, TNFα, IL-6, and MIP1 in monocytes [97].
    HMGB1 can be also inhibited by blocking the heterocomplex with CXCL12 (HMGB1·CXCL12) impairing the formation of HMGB1/CXCL12/CXCR4 and subsequent chemoattraction, with small molecules such as glycyrrhizin [98] and salicylates such as diflunisal, salicylic acid [99], 5,5′-methylenedi-2,3-cresotic acid (MCA) [100]; and more recently, pamoic acid, a solubilizer commonly used in drug formulations due to its lack of gastrointestinal absorption [101]. Drugs currently used to treat SSc, such as MTX [102], can also bind to HMGB1 at two independent sites, inhibiting the HMGB1/RAGE interaction. Consequently, they can at least partially attribute its effects to this pathway.
  B. Inhibition of HMGB1 extracellular release. Other compounds, such as ethyl pyruvate, a common food additive, can inhibit HMGB1 active release in lymphoma cells [103].
  C. Antibody binding/blockage of HMGB1. Polyclonal and, more recently, monoclonal anti-HMGB-1 antibodies have been developed. Studies on SLE have yielded conflicting results, with some showing amelioration of the progression of the lupus phenotype (MRL/lpr and BXSB mice) [104, 105]. In contrast, another study found no effects in disease progression in MRL/lpr mice [106].
  D. Sham-HMGB1 BoxA competitive inhibition. The A box domain alone can bind to TLR2/4 and RAGE without triggering downstream inflammatory responses and can be used as a HMGB1 potent competitive inhibitor [107]. Consequently, HMGB1 has a high potential to become a SSc drug target for treating SSc since it plays a crucial role in inflammation, vasculopathy, and fibrosis, and there are several known drugs and preclinical compounds that bind and antagonize HMGB1 directly and indirectly. Furthermore, once methods for quantifying free HMGB1 serum levels are standardized, they can serve as their own biomarker. A list of selected inhibitors with potential clinical use is summarized in Table 1.

**Table 1:** HMGB1 targeted therapy

Drugs	Mechanism of Action	Clinical Development	Effects	References
Diflunisal, Glycyrrhizin, Pamoic acid.	Inhibition of CXCL12, CXCR4, TLR interaction with HMGB1.	Glycyrrhizin is approved in Asian countries for chronic hepatitis C; Diflunisal is used as NSAID.	Reduces inflammation	[98–101]
Ethyl pyruvate	Inhibition of HMGB1 extracellular release.	In vitro studies show decreased HMGB1 release.	Inhibits HMGB1 release from B-cell lymphoma cells.	[103]
Anti-HMGB1 Antibodies.	Polyclonal and monoclonal blockade of HMGB1.	Preclinical data in SLE and RA models.	Improved RA and mixed data in SLE animal models.	[104–106]
SB17170. (small molecule)	Unknown, blocking HMGB1 activation of myeloid cells.	Phase II trials for Idiopathic Pulmonary Fibrosis (IPF).	Ongoing Phase IIa clinical trial.	[108]
Sham HMGB1 Box A.	Decoy binding TLR2/4 and RAGE.	Preclinical data in RA mouse models.	Reduces inflammation in RA animal models.	[107]
MTX, Soluble RAGE.	Inhibition of HMGB1-RAGE interaction.	It is widely used to treat SSc and RA. Reduces arterial calcification in animal models.	Reduces inflammation and calcification in preclinical models.	[102]
2. **Inhibition of SOX9.**
  On the other hand, the complete inhibition of the SOX-9 transcription factor is not lethal, and a large number of potential inhibitors have been identified [109] with potential therapeutic applications in fibrotic diseases, cancer, and other pathologies. Calmodulin antagonists such as KN-93, calmidazolium, and W-7 block CaMKIIs and consequently can block CaMKII-mediated SOX-9 activation [110–112]. BRD4 inhibitors such as JQ1 suppress Sox9 transcription but face pharmacokinetic limitations, whereas newer bromodomain inhibitors (e.g., ODM207) have shown early clinical data [89]. Gene silencing with Sox9-specific siRNA/shRNA has been used in animal models, reducing tumorigenicity in cancer models. A list of SOX-9 inhibitors and their mechanism of action is shown in Table 2.

**Table 2:** SOX9 targeted therapies

Drug	Mechanism of Action	Clinical Development	Effects	References
Calmodulin antagonist (Calmidazolium)	Calmodulin antagonist. Blocks CaMKII-mediated SOX9 activation Calmidazolium: conformational stabilization of CaM domains	Calmidazolium: In vitro and in animal models, inhibits cancer cell growth. Unspecific	Inhibits SOX9 activation. Reduce tumorigenicity in cancer models.	[111, 112]
BRD4 inhibitors (JQ1)	BRD4 inhibitors. Suppresses Sox9 transcription by inhibiting bromodomain proteins.	JQ1:In vitro data for cancer models.	JQ1 Suppresses SOX9 transcription in vitro with potential for use in cancer, fibrosis, and other pathologies.	[89]
Curcumin / Resveratrol	Multi-targeted modulation of signaling and transcription. Downregulate SOX9 indirectly.	Dietary supplements / investigational.	Modest SOX9 reductions in cell/animal models; anti-inflammatory and anti-proliferative effects reported.	[110]
CDK7 / Super-enhancer inhibitors (THZ2)	Covalent inhibitor targeting the super-enhancer (SE) component CDK7.	In vitro and in vivo assays.	Exert antitumor effects and act synergistically with DNA methylation drugs.	[113]
microRNAs targeting SOX9 (miR-539-3p, miR-101-3p)	Inhibition of SOX9 expression.	In vitro assays for miR539-3p and miR-101-3p.	miR539-3p Inhibits Chondrogenic Differentiation in Adipose stem-cell miR-101-3p Promotes calcification in HAVICs.	[114, 115]
siRNA / shRNA against SOX9	Direct knockdown of SOX9 mRNA.	In vitro studies.	Inhibition of malignant cell growth.	[116]

Whereas HMGB1 and Sox9 inhibitors are still in the early stages of discovery and development, they have the potential to halt multiple SSc pathways, including fibrosis, inflammation, and vasculopathy, and are promising antifibrotic agents for SSc.

Despite multiple potential mechanisms to interfere with HMG-related proteins, many compounds have shown mild inhibition and non-specific binding, and are known to be barriers to clinical development. This is likely driven by the flexible, solvent-exposed structure of HMG, lacking deep enough hydrophobic pockets for high-affinity drug binding [117]. However, recent advances in structural biology, computational modeling, and chemical approaches, including in silico docking studies of hydrophobic pocket interactions and the development of novel small-molecule inhibitors that specifically target these pockets, are likely to drive the development of optimized inhibitors and fuel clinical development. In fact, SB17170, a small-molecule prodrug HMGB1 inhibitor, has just initiated a Phase 2 clinical trial in patients with Idiopathic Pulmonary Fibrosis [108]. 

In addition, the emerging field of proteolysis-targeting chimeras (PROTACs), which target intracellular and intranuclear proteins for proteasomal degradation, opens the possibility of targeting HMG proteins with this approach [118].

## Concluding remarks

SSc is a highly complex autoimmune disease of unknown etiology, causing progressive cutaneous and multiple organ fibrosis and severe vasculopathy. Despite the severity of the disease, there is no curative therapy at present. However, further knowledge of its pathogenesis is expected to yield effective therapeutic approaches. This review describes recent investigations that demonstrate a key role of transcription factors sharing an HMG motif, such as SOX9 and HMGB1, in systemic sclerosis and suggests a possible role in SSc pathogenesis beyond their function as transcription factors, encompassing multiple aspects of the disease. This review further proposes that the HMG motif may be a valuable target for developing novel treatments for this serious autoimmune pathological condition.

## Data Availability

All available data are included in the manuscript.
